# Case Report: Two siblings with a novel homozygous SLC18A2 variant causing parkinsonism-dystonia-2: a case series from Saudi Arabia

**DOI:** 10.3389/fgene.2026.1812336

**Published:** 2026-05-21

**Authors:** Meshal Almutair, Wejdan Hakami

**Affiliations:** Division of Pediatric Neurology, Department of Pediatrics, Prince Sultan Military Medical City, Riyadh, Saudi Arabia

**Keywords:** monoamine neurotransmitter disorder, oculogyric crises, parkinsonism-dystonia-2, SLC18A2 gene, VMAT2

## Abstract

**Background:**

Monoamine neurotransmitter disorders are rare, early-onset neurological conditions that frequently mimic cerebral palsy or epileptic encephalopathies, resulting in diagnostic delay. Parkinsonism-dystonia-2 (PKDYS2), caused by biallelic variants in *SLC18A2*, encodes vesicular monoamine transporter 2 (VMAT2), which is essential for dopamine and serotonin vesicular storage and release. The disorder is characterized by global developmental delay, parkinsonism, dystonia, and autonomic dysfunction.

**Methods:**

This retrospective case report describes two siblings from a consanguineous family diagnosed with PKDYS2. Clinical, neuroimaging, and genetic data were collected from medical records, with longitudinal follow-up to assess disease progression and treatment response. Whole-exome sequencing (WES) was performed, and variants were analyzed using standard bioinformatics pipelines. A homozygous splice-site variant in *SLC18A2* (c.1122 + 2T>C) was identified, classified as likely pathogenic according to ACMG/ClinGen criteria, and confirmed by Sanger sequencing with parental segregation.

**Results:**

Both siblings presented in early infancy with hypotonia, developmental delay, dystonia, and oculogyric crises, with normal neuroimaging. WES identified a homozygous *SLC18A2* splice-site variant (c.1122 + 2T>C) in both patients. Pramipexole resulted in partial improvement in one patient but was poorly tolerated in the other. Alternative therapies, including clonidine and trihexyphenidyl, provided limited symptomatic benefit. Both patients demonstrated severe and persistent neurodevelopmental impairment at follow-up.

**Conclusion:**

This case report identifies a previously unreported *SLC18A2* splice-site variant (c.1122 + 2T>C) in two siblings with a severe neurodevelopmental phenotype characterized by hypotonia, dystonia, autonomic dysfunction, and parkinsonian features. These findings highlight the key role of genetic testing in establishing the diagnosis, avoiding ineffective treatments, and guiding management. Early genetic evaluation should be considered in children with early-onset movement disorders.

## Introduction

Monoamine neurotransmitter disorders are a group of rare, heterogeneous neurological conditions that typically present in early childhood (Ng et al., 2015). These disorders often mimic other neurological diseases, such as cerebral palsy or hypoxic-ischemic encephalopathy, leading to potential misdiagnosis (Kurian et al., 2011). The coexistence of dyskinetic movements and autonomic dysfunction should prompt consideration of a neurotransmitter disorder and warrant early genetic evaluation for diagnostic confirmation.

Biallelic loss-of-function (LoF) variants in *SLC6A3* (OMIM 126455), which encodes the dopamine transporter, cause infantile-onset parkinsonism-dystonia 1 (OMIM 613135), also known as dopamine transporter deficiency syndrome (DATS), the first monoamine transportopathy to be identified (Kurian et al., 2009).

Another critical gene, *SLC18A2* (OMIM 193001), encodes brain vesicular monoamine transporter 2 (VMAT2), which facilitates the loading of dopamine and serotonin into synaptic vesicles for transport to the cell membrane and subsequent release (Fon et al., 1997). Biallelic dysfunction of *SLC18A2* results in brain monoamine vesicular transport disease, known as parkinsonism-dystonia-2 (PKDYS2) (OMIM 618049). PKDYS2 is an autosomal-recessive disorder characterized by global developmental delay, parkinsonism, dystonia, and autonomic dysfunction (Rilstone et al., 2013). First described in 2013 in eight children from an extended consanguineous Saudi Arabian family, PKDYS2 presents with a wide spectrum of monoaminergic symptoms. These include dystonia, parkinsonism, oculogyric crises (associated with dopaminergic defects), sleep and mood disorders (linked to serotonergic dysfunction), excessive sweating, temperature instability, ptosis, and postural hypotension (resulting from adrenergic and noradrenergic abnormalities) (Rilstone et al., 2013). This complexity underscores the importance of early recognition and accurate diagnosis for effective management. This report describes two siblings with PKDYS2 harboring a previously unreported splice-site variant in *SLC18A2*, and provides a clinical description of their presentation, diagnostic evaluation, and observed treatment responses.

## Methods

### Study design and data collection

We describe two siblings from a consanguineous family diagnosed with PKDYS2. Detailed clinical, neuroimaging, and genetic data were retrospectively collected from medical records. Both patients were followed longitudinally to assess clinical evolution, therapeutic response, and complications.

### Genetic analysis

Genomic DNA was extracted from peripheral blood of both siblings and parents, and whole-exome sequencing (WES) was performed using the Roche KAPA HyperExome enrichment kit and MGI DNBSEQ platform. Reads were aligned to the GRCh37/hg19 reference genome using BWA (v0.7.17), and variant calling was conducted with GATK (v4.1.9.0). Variants were annotated and filtered using population and disease databases, prioritizing rare variants consistent with autosomal recessive inheritance.

A homozygous canonical splice-site variant in SLC18A2 (NM_003054.4:c.1122 + 2T>C) was identified. The variant affects the canonical splice donor site and is predicted to result in loss of function. It was absent from population databases including gnomAD. Variant interpretation followed ACMG/ClinGen guidelines and was classified as likely pathogenic. Sequencing quality metrics showed a mean coverage depth of 239×, with 99.7% of target bases covered at ≥20×.

The variant was confirmed by Sanger sequencing, with segregation analysis demonstrating heterozygosity in both parents.

## Case presentations

### Case 1

**FIGURE 1:**
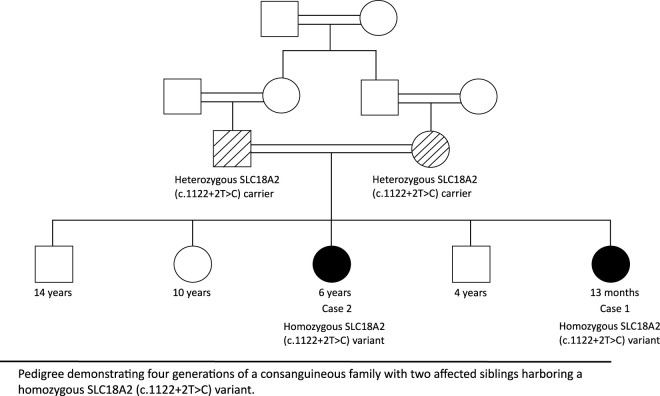
Pedigree of a consanguineous family with an SLC18A2 variant.

A 13-month-old girl, born at term following an uncomplicated pregnancy and delivery. She is the product of a consanguineous marriage (first cousins) (Figure 1). Early developmental milestones were initially appropriate. At 4 months of age, the patient developed progressive hypotonia accompanied by motor regression. Around the same time, she began to exhibit recurrent episodes of generalized dystonic posturing associated with upward gaze deviation, occurring with preserved awareness, consistent with oculogyric crises. She also demonstrated autonomic features, including excessive sweating, as well as oro-lingual incoordination affecting feeding. Initial neurological evaluation demonstrated generalized hypotonia and global developmental delay. At the referring hospital, seizures were initially suspected, and levetiracetam was initiated; however, no clinical improvement was observed, prompting referral to our center.

On assessment at our institution, the patient was an interactive child with generalized severe hypotonia involving both axial and appendicular musculature, with a persistent head lag. Intermittent dystonic posturing with oculogyric crises was noted. Electroencephalogram (EEG) captured the clinical events without associated epileptiform activity, leading to discontinuation of antiseizure therapy. Brain magnetic resonance imaging (MRI) was unremarkable.

Given the clinical presentation, a neurotransmitter disorder was considered. Empirical treatment with levodopa–carbidopa was initiated at a dose of up to 8 mg/kg/day, divided into three doses, but no clinical improvement was observed. At higher doses, worsening of dystonic symptoms was reported, prompting discontinuation of the medication. Although cerebrospinal fluid (CSF) neurotransmitter analysis was planned, it could not be performed due to technical limitations.

At 5 months of age, in view of a similarly affected older sibling, genetic testing was pursued. WES identified a homozygous canonical splice-site variant in SLC18A2 (NM_003054.4:c.1122 + 2T>C), classified as likely pathogenic, supporting the diagnosis of PKDYS2.

Treatment with pramipexole was initiated at 0.005 mg/kg/day, divided into three daily doses, and subsequently titrated to 0.01 mg/kg/day. This was associated with a marked clinical improvement in dystonic posturing and oculogyric crises, reflected by a reduction in the frequency and duration of dystonic episodes, as well as improved spontaneous motor activity and feeding. Given the patient’s young age, formal quantification using standardized dystonia rating scales was not feasible; therefore, treatment response was assessed based on serial clinical evaluations and caregiver-reported improvement.

At the most recent follow-up (13 months of age), the patient continued to exhibit significant global developmental delay, absent head control and marked oropharyngeal dysfunction with impaired swallowing, necessitating gastrostomy tube feeding. Despite these significant motor impairments, she retained the ability to visually fix, track and exhibited meaningful social interaction with family members. No significant medication-related adverse effects were observed during follow-up. Supportive management, including nutritional support and rehabilitation (physiotherapy and occupational therapy) were initiated.

### Case 2 (older sibling)

A 6-year-old girl, born at term following an uncomplicated pregnancy and delivery, presented at 5 months of age with generalized hypotonia, failure to thrive, and recurrent episodes of dystonic posturing associated with oculogyric crises.

These events were initially presumed to be epileptic in nature, and the patient was treated sequentially with multiple antiseizure medications, including valproic acid, levetiracetam, and clonazepam; however, no clinical improvement was observed. An interictal EEG demonstrated generalized slowing for age without epileptiform discharges. MRI was normal.

Over time, she developed severe global developmental delay and intellectual disability and became dependent on nasogastric tube feeding.

The patient was subsequently referred to our center at 5 years of age following the diagnosis of her younger sibling. At presentation, she demonstrated poor interaction and was non-verbal, with moderate-to-severe intellectual disability. No oculogyric crises were observed. Neurological examination revealed axial hypotonia with appendicular dystonia, in addition to bradykinesia, hypomimia, and reduced spontaneous movements. She was non-ambulatory.

WES identified the same homozygous canonical splice-site variant in *SLC18A2* (NM_003054.4:c.1122 + 2T>C), classified as likely pathogenic, thereby supporting the diagnosis of PKDYS2.Based on this finding, antiseizure medications were discontinued, and treatment with pramipexole was initiated at 0.01 mg/kg/day. However, therapy was complicated by adverse effects, including facial dyskinesia and insomnia, leading to its discontinuation.

Subsequently, the patient was started on levodopa–carbidopa; however, even at a low dose, she developed orofacial dyskinesia accompanied by irritability and behavioral changes, leading to its discontinuation. Clonidine was then initiated, with mild symptomatic improvement in dystonic features. Trihexyphenidyl was later introduced during follow-up for additional symptomatic management.

At the most recent follow-up (6 years of age), she remained nonverbal, severely developmentally delayed, and fully dependent for activities of daily living, with persistent dystonia and parkinsonian features. Supportive care, including nutritional support and rehabilitation therapies, was ongoing.

**FIGURE 2:**
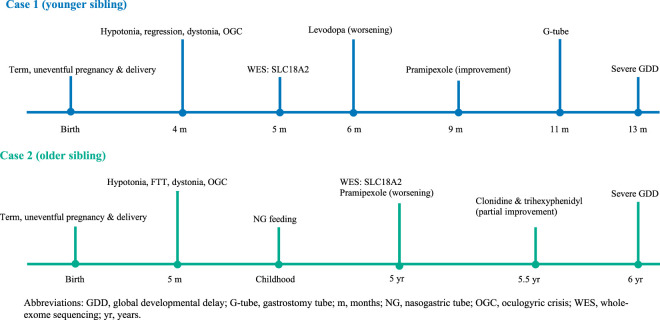
Clinical timelines of two siblings with SLC18A2-related PKDYS2.

A comparative summary of the phenotypic presentation, investigations, and treatment responses of both patients is presented in Table 1, while Figure 2 illustrates the clinical timelines of the two siblings with SLC18A2-related PKDYS2.

**TABLE 1:** Phenotypic presentation of two siblings with SLC18A2-related PKDYS2.

Clinical feature	Case 1 (younger sibling)	Case 2 (older sibling)
Birth history	Born at term following an uncomplicated pregnancy and delivery	Born at term following an uncomplicated pregnancy and delivery
Early presentation (4–5 months)	Hypotonia, motor regression, dystonia, oculogyric crises, and autonomic symptoms	Hypotonia, failure to thrive, oculogyric crises, and dystonia
EEG findings	Normal ictal EEG	Interictal EEG showing generalized slowing for age without epileptiform discharges
Brain MRI findings	Normal	Normal
Levodopa trial	Initiated at 6 months → worsening dystonia	Initiated at 5 years → orofacial dyskinesia, irritability, and behavioral disturbances
Genetic testing	WES at 5 months → SLC18A2 variant identified	WES at 5 years → same SLC18A2 variant identified
Dopamine agonist (pramipexole)	Initiated at 9 months → clinical improvement in dystonia and autonomic features	Initiated at 5 years → dyskinesia and insomnia → discontinued
Alternative therapies	—	Clonidine and trihexyphenidyl → partial improvement in dystonia
Feeding status	Dysphagia requiring gastrostomy tube placement at 11 months	Nasogastric tube feeding since 2 years of age
Current status	Global developmental delay with absent head control; able to fix and follow visually	Severe developmental delay, intellectual disability, nonverbal, with persistent parkinsonian features

Abbreviations: EEG, electroencephalogram; MRI, magnetic resonance imaging; PKDYS2, Parkinsonism–dystonia type 2; WES, whole-exome sequencing.

## Discussion

Variants in *SLC18A2* are associated with a neurodevelopmental disorder characterized by global developmental delay, hypotonia, dystonia, parkinsonism, and autonomic dysfunction, including temperature dysregulation, abnormal sweating, hypersalivation, gastrointestinal dysmotility, and oculogyric crises, reflecting the widespread impact of monoaminergic dysfunction (Saida et al., 2026).

A characteristic clinical feature of *SLC18A2*-related disease is the coexistence of extrapyramidal manifestations and autonomic dysfunction in the setting of structurally normal neuroimaging (Saida et al., 2026). This constellation should prompt consideration of a neurotransmitter disorder, particularly in infants presenting with hypotonia and paroxysmal dystonic events. Although neuroimaging is typically normal, atypical findings have been reported. For example, Almuqbil et al. (2024) described a Saudi cohort harboring a homozygous *SLC18A2* variant (c.710C>A; p.Pro237His), with bilateral symmetric T2 hyperintensity and restricted diffusion involving the dorsal brainstem and pons (Almuqbil et al., 2024).

Although CSF neurotransmitter analysis was not performed in our patients due to technical limitations, prior studies have shown that CSF profiles may be normal in individuals with PKDYS2, thereby limiting its diagnostic sensitivity (Saida et al., 2026). These findings suggest that biochemical testing may not reliably detect underlying monoaminergic disorders, supporting the central role of genetic testing in establishing a molecular diagnosis. Other primary neurotransmitter disorders, such as aromatic L-amino acid decarboxylase (AADC) deficiency, sepiapterin reductase (SPR) deficiency and tyrosine hydroxylase (TH) deficiency, were considered in the differential diagnosis. However, the identification of a likely pathogenic *SLC18A2* variant supports the diagnosis in the appropriate clinical context. Comprehensive exome analysis did not identify other pathogenic or likely pathogenic variants that could explain the phenotype. Variants in genes associated with neurotransmitter disorders (e.g., AADC, SPR, and TH) were specifically reviewed and excluded based on variant filtering criteria, inheritance pattern, and clinical correlation.

Beyond supporting the diagnosis, genetic testing enables clinicians to discontinue ineffective therapies, such as antiseizure medications, and to initiate more targeted treatments (Rilstone et al., 2013). In our patients, genetic analysis identified a homozygous splice-site variant in *SLC18A2* (c.1122 + 2T>C), classified as likely pathogenic according to ACMG criteria. In silico splice prediction tools, including SpliceAI, MaxEntScan, and Human Splicing Finder, consistently predicted disruption of the canonical donor splice site. The variant is predicted to disrupt the canonical splice donor site, consistent with a LoF mechanism. Specifically, the c.1122 + 2T>C variant affects the highly conserved +2 position and is predicted to result in aberrant splicing, potentially leading to exon skipping or activation of a cryptic splice site, causing a frameshift and premature termination codon with subsequent nonsense-mediated mRNA decay. According to ACMG/AMP guidelines, this variant was classified as likely pathogenic based on PVS1 (predicted loss-of-function in a gene where LoF is a known mechanism), PM2 (absence from population databases), and PP1 (segregation with disease in affected siblings). This report therefore describes a previously unreported variant in *SLC18A2*, rather than establishing new genotype–phenotype correlations. Although functional validation was not performed, the variant is considered causative based on its canonical splice-site location, predicted loss-of-function effect, absence in population databases, segregation within the family, and strong phenotypic concordance with previously reported SLC18A2-related disease.

Genotype–phenotype correlations have been suggested in *SLC18A2*-associated disorders, with certain variants appearing to be associated with differing degrees of disease severity. The p.Pro237His variant has been reported in association with more severe phenotypes, whereas variants such as p.Pro387Leu and p.Ile43Phe have been described in individuals with comparatively milder clinical presentations (Saida et al., 2026). These observations highlight the clinical heterogeneity of the disorder and underscore the importance of accumulating additional case data to better define potential genotype–phenotype relationships. Intrafamilial phenotypic variability, as observed in our patients, may reflect the influence of genetic modifiers, epigenetic factors, environmental exposures, and variability in residual protein function. In monoaminergic disorders, differences in compensatory neurotransmitter pathways and receptor sensitivity may further contribute to variability in clinical severity and treatment response.

Treatment response in SLC18A2-related PKDYS2 remains heterogeneous and poses significant therapeutic challenges. Dopamine agonists, such as pramipexole or bromocriptine, may provide symptomatic benefit, whereas levodopa often demonstrates limited efficacy and may exacerbate dystonia or induce hyperkinetic movements (Saida et al., 2026; Zhai et al., 2021). In our cases, levodopa was not associated with clinical benefit and instead appeared to exacerbate hyperkinetic movements, including worsening dystonia in Case 1 and the emergence of orofacial dyskinesia with behavioral disturbances in Case 2. This observation is consistent with previous reports suggesting that levodopa may have limited efficacy or even paradoxical effects in VMAT2 deficiency, likely due to impaired vesicular monoamine storage and consequent accumulation of cytosolic dopamine (Ng et al., 2015; Rilstone et al., 2013).

In contrast, pramipexole demonstrated variable tolerability and efficacy. One patient showed improvement in dystonic features, oculogyric crises, and autonomic dysfunction, whereas the other developed dose-limiting adverse effects, including dyskinesia and insomnia. Dopamine agonists may provide symptomatic benefit by bypassing presynaptic dopamine handling defects and directly stimulating postsynaptic receptors (Kurian et al., 2009). However, the variability in response observed in our patients underscores the need for careful dose titration and close monitoring for adverse effects.

Symptomatic therapies, including clonidine and trihexyphenidyl, were associated with partial improvement in dystonic features in the more severely affected sibling. These findings further support the role of individualized, symptom-targeted management strategies in this disorder. Notably, the two siblings in our report exhibited differential clinical severity and treatment response despite sharing the same homozygous SLC18A2 variant, highlighting marked intrafamilial phenotypic variability.

Although the observed improvement with pramipexole in one patient may partially reflect natural disease fluctuation, the temporal association with treatment initiation supports a therapeutic effect. Collectively, these observations emphasize the complexity of treatment in PKDYS2 and highlight the importance of individualized therapeutic strategies guided by clinical response and tolerability.

On follow-up, both patients exhibited severe and persistent neurodevelopmental impairment. Case 1 retained limited visual tracking and social interaction despite profound motor dysfunction, whereas Case 2 remained fully dependent for all activities of daily living, with severe intellectual disability and persistent parkinsonian features. These findings are broadly consistent with previously reported phenotypes and highlight the substantial functional burden associated with this disorder (6,7).

This study has several strengths, including the identification of a previously unreported SLC18A2 variant and detailed longitudinal clinical characterization. However, certain limitations should be acknowledged, including the absence of functional validation, lack of cerebrospinal fluid neurotransmitter analysis, small sample size, and relatively short follow-up, which limit the evaluation of long-term outcomes and treatment durability.

From the family’s perspective, the diagnostic journey was prolonged and emotionally challenging, with multiple ineffective treatments prior to establishing a molecular diagnosis. Identification of the underlying genetic cause provided clarity, guided management decisions, and enabled appropriate genetic counseling regarding recurrence risk.

Collectively, these findings underscore the importance of considering SLC18A2-related disorders in infants presenting with early-onset dystonia and autonomic dysfunction, even in the context of normal neuroimaging and inconclusive biochemical investigations.

## Conclusion

This case series describes a previously unreported SLC18A2 variant (c.1122 + 2T>C) identified in two siblings from a consanguineous Saudi family, both presenting with a severe neurodevelopmental disorder characterized by global developmental delay, hypotonia, dystonia, and autonomic dysfunction, with additional parkinsonian features observed in one patient.

These findings highlight the important role of genetic testing in establishing the diagnosis, facilitating the discontinuation of ineffective therapies, and guiding targeted management. Early integration of genetic testing into the diagnostic evaluation of children with early-onset movement disorders may influence clinical decision-making and improve patient care. Given the autosomal recessive inheritance, genetic counseling is essential, as parents are obligate carriers with a 25% recurrence risk in future pregnancies. Cascade carrier testing for at-risk relatives and discussion of reproductive options, including prenatal diagnosis and preimplantation genetic testing, are recommended. Further studies are warranted to better define genotype–phenotype correlations and to establish evidence-based management strategies for PKDYS2.

## Data Availability

The data supporting the findings of this study are available from the corresponding author upon reasonable request.
